# Intracellular pH regulation in mantle epithelial cells of the Pacific oyster, *Crassostrea gigas*

**DOI:** 10.1007/s00360-020-01303-3

**Published:** 2020-08-20

**Authors:** Kirti Ramesh, Marian Y. Hu, Frank Melzner, Markus Bleich, Nina Himmerkus

**Affiliations:** 1 GEOMAR Helmholtz Centre for Ocean Research, Kiel, 24105 Germany; 2 Department of Biological & Environmental Sciences, Gothenburg University, Gothenburg, Sweden; 3 Institute of Physiology, Christian Albrechts University Kiel, 24118 Kiel, Germany

**Keywords:** Mollusc, Calcification, Acid–base, Ion transport proteins

## Abstract

Shell formation and repair occurs under the control of mantle epithelial cells in bivalve molluscs. However, limited information is available on the precise acid–base regulatory machinery present within these cells, which are fundamental to calcification. Here, we isolate mantle epithelial cells from the Pacific oyster, *Crassostrea gigas* and utilise live cell imaging in combination with the fluorescent dye, BCECF-AM to study intracellular pH (pH_i_) regulation. To elucidate the involvement of various ion transport mechanisms, modified seawater solutions (low sodium, low bicarbonate) and specific inhibitors for acid–base proteins were used. Diminished pH recovery in the absence of Na^+^ and under inhibition of sodium/hydrogen exchangers (NHEs) implicate the involvement of a sodium dependent cellular proton extrusion mechanism. In addition, pH recovery was reduced under inhibition of carbonic anhydrases. These data provide the foundation for a better understanding of acid–base regulation underlying the physiology of calcification in bivalves.

**Electronic supplementary material:**

The online version of this article (10.1007/s00360-020-01303-3) contains supplementary material, which is available to authorized users.

## Introduction

Shells of the Pacific oyster, *Crassostrea gigas,* a mollusc species with enormous economic and ecological value (Zhang et al. 2012) are characterized by the presence of two calcareous valves. In adult *C. gigas* molluscs, shell formation occurs under the control of the mantle tissue, where mantle epithelial cells take part in the transport of calcification substrates (Gong et al. 2008) (Ca^2+^ and HCO_3_^−^). Recently, Sillanpaa et al. (2018) reported that up to 60% of the Ca^2+^ transport in *C. gigas* mantle epithelia occurs via the transcellular pathway. However, it is unknown at present by which pathways bivalve molluscs control the precipitation of the calcareous shell. Since proton production is a by-product of mineral formation from the bicarbonate (HCO_3_^−^) substrate (Zeebe and Wolf-Gladrow 2001), specific mechanisms have to be present in calcifying molluscs to minimize fluctuations in intracellular pH (pH_i_). For calcification to take place, mantle epithelial cells would be responsible for generating and maintaining a highly regulated microenvironment. This makes intracellular pH homeostasis intricately linked to biomineralization as HCO_3_^−^ and Ca^2+^ import will require the export or buffering of protons to maintain pH_i_.

Regulation of pH_i_ is a key aspect of cell physiology and cells make use of evolutionarily conserved membrane-bound transport proteins or intracellular buffering to accomplish stable pH_i_ (Laurent et al. 2014). Membrane-bound transport proteins can achieve proton extrusion (sodium/hydrogen exchangers (NHEs), V-type proton ATPases), bicarbonate uptake (sodium driven bicarbonate transporters) and most importantly generate the electrochemical gradient driving pH_i_ regulation (sodium–potassium ATPases). In addition to pH_i_ regulation, these membrane-transporters are also crucial for calcification by providing the substrates necessary through cellular ion transport. This occurs via primary (calcium-ATPases) and secondary (sodium/calcium exchangers and sodium driven chloride/bicarbonate exchangers) pathways. Additionally, membrane transporters also aid in the removal of proton by-products from the calcification substrate HCO_3_^−^ (Ramesh et al. 2019). Molecular evidence for the presence of such transporters in molluscs comes from cloning of calcium transporting proteins within the mantle tissue in the pearl oyster, *Pinctada fucata* and the observed expression of all of the above-mentioned pH_i_ regulatory proteins in several mollusc species (Wang et al. 2008; Zhang et al. 2012; De Wit et al. 2018). Maintenance of stable pH_i_ has been demonstrated for a range of muscle types including cardiac myocytes and adductor and byssus retractor muscles (Ellington 1983, 1993; Zange et al. 1990). In *C. gigas*, pH_i_ regulation in such non-calcifying tissues has been shown to be dissimilar to the mantle, as cardiac tissues exhibit intrinsically higher intracellular non-bicarbonate, non-phosphate buffer capacities (Michaelidis et al. 2005). Regulation of pHi can also be achieved via intracellular modulation of bicarbonate concentrations via carbonic anhydrases and a range of other proton equivalent exchange processes. Carbonic anhydrases can produce HCO_3_^−^ from intracellular CO_2_ and their role in biomineralisation has been studied in a range of molluscs (Medakovic 2000; Marie et al. 2008). However, there is limited information regarding the role of carbonic anhydrases in pH_i_ regulation, particularly in calcifying tissue.

Characterization of the ability of bivalve mantle cell acid–base regulatory mechanisms during induced stress, is rudimentary, although knowledge of the pH_i_ control mechanisms in bivalve models such *C. gigas* is particularly important in the context of anthropogenic seawater acidification. Anthropogenic seawater acidification is accompanied with elevated dissolved carbon dioxide which consequently alters extracellular acid–base homeostasis (Zlatkin and Heuer 2019; Melzner et al. 2020). Similar to many molluscs, oysters are considered to be weak extracellular acid–base regulators and can only partially compensate for induced acidosis (Dwyer and Burnett 1996). Therefore, the relationship between seawater acidification, and pH_i_ regulation is intricate and our ability to accurately predict the response of calcifying bivalves to seawater acidification is severely hindered by our limited understanding of the cellular mechanisms driving pH homeostasis in these organisms.

Here, we use primary mantle cell cultures to investigate the previously uncharacterized pH_i_ regulatory capacity of mantle epithelial cells in *C. gigas* using live cell imaging and the pH-sensitive fluorescent probe, 2′,7′-bis-(2-carboxyethyl)-5-(and-6)-carboxyfluorescein acetoxymethyl ester (BCECF-AM). Furthermore, we utilize pharmaceutical blockers and modifications in ion composition to investigate the role of key ion transport pathways to provide crucial information on the mechanisms of pH_i_ regulation in molluscan calcifying tissue.

## Materials and methods

### Oyster collection and maintenance

Adult aquaculture raised *C. gigas* were purchased from Dittmeyer’s Austern Compagnie GmbH (‘Sylter Royal’, List, Germany) and delivered over night to GEOMAR Helmholtz Centre for Ocean Research Kiel. Animals were utilized for experiments within 30 days following arrival. Animals were maintained at a temperature of 11 °C and pH_NBS_ of 8.0 ± 0.1 in an aerated, re-circulating seawater system without feeding in seawater prepared using Instant Ocean to a salinity of 31 ± 2 psu. Water in the culture system was exchanged on a weekly basis. Ammonium concentrations were tested using a JBL NH_4_^+^ aquaria kit and maintained below a concentration of 0.05 mg/L.

### Mantle cell culture

Primary mantle cells were cultured according to Gong et al. (2008) with slight modifications. Briefly, oysters were dissected, and pallial mantle tissue as described in Gong et al. (2008) was excised for cell culture. The mantle tissue was sanitized for 20 min in an artificial seawater solution (ASW, Supplementary Table 1) containing 0.5 mg/ml streptomycin, 500 IU/ml penicillin,100 IU/ml gentamicin, and 2 μg/ml nystatin. After rinsing 3 times in a calcium and magnesium free ASW (Supplementary Table 1), the pallial mantle tissue was minced into small fragments (approx. 2 mm diameter) and these fragments were planted onto the center of lysine coated glass coverslips (Eydam, Germany), placed into sterile petridishes. Cell cultures were maintained in a culture medium that is detailed in Supplementary Table 2. Cells of interest were allowed to migrate out of explants for 24 h, explants were removed and sedentary culture cells remaining on the coverslips were used for microfluorimetry. All reagents were purchased from Sigma-Aldrich unless otherwise specified.

### Preparation of solutions

Artificial seawater (ASW) solutions were prepared according to Zeebe and Wolf-Gladrow (2001) (Supplementary Table 1). Osmolality (1104 ± 5 mOsm kg^−1^) and salinity (31 ± 2 psu) were selected to match the seawater values in the culture system (1113 ± 8 mOsm kg^−1^). Inhibitors were dissolved in DMSO and added at final concentrations of 20 µM (ethylisopropyl amiloride, EIPA) and 1 mM (acetazolamide, ACZM) to ASW. DMSO concentrations did not exceed 0.1%.

### BCECF dye loading

To measure mantle epithelial cell pH_i_, cover slips containing cell cultures were affixed to glass perfusion chambers (Supplementary Fig. 1) using a hydrophobic, silicone gel and bathed in ASW with a final BCECF-AM concentration of 10 μM at 19 °C for 30 min in the dark. Following dye loading, cells that were firmly attached to the coverslip were used for measurements. The flow rate of the perfusion system was 1–2 mL min^−1^ and experiments were performed at 19 °C.

### Microfluorimetry

Microfluorimetric measurements were performed on an inverted microscope (Zeiss Axio Observer. D1) equipped with a 40 × objective (Zeiss) and a CoolSNAP HQ^2^ CCD camera (Photometrics, USA). The dye was excited alternatively at two wavelengths, 486 nm and 439 nm (± 10 nm bandwidth) for 24 and 60 ms, respectively. Emission was recorded at 525 nm and fluorescence was monitored with the imaging system Visitron. The ratio of the emission intensities at the two excitation wavelengths over mantle cell was calculated, following background subtraction of camera offset using the software Metafluor 7.6.1. From each coverslip, the recordings of one to six mantle epithelial cells were collected and averaged. For each treatment, between 4 and 7 individual oysters were used as biological replicates. The ionophore nigericin was used to calibrate pH_i_ of mantle cells as previously described by Stumpp et al. (2012). Mantle cells were exposed to 10 μM nigericin in the presence of 160 mM potassium [K^+^] at pH 6.5, 7.0, 7.5 and 8.0. This K^+^ concentration was chosen to be in the range of intracellular [K^+^] reported for marine molluscs (Potts 1958; Ellington 1993). The calibration curve allowed calculation of the relationship between recorded emission ratio of BCECF and the corresponding pH_i_. For pH_i_ recovery experiments, mantle cells were exposed to ASW for 10 min followed by a 20 mM NH_3_/NH_4_^+^ pulse. Alkalosis compensation rates were calculated as the slope during this ammonia prepulse phase and are indicative of the rate of active pHi acidification during the NH_3_/NH_4_^+^ prepulse (Table 1). Acidosis was consecutively induced by the washout of NH_3_/NH_4_^+^ using the following solutions: ASW as control condition and 5 mM Na^+^, low HCO_3_^−^ or ASW plus inhibitors (see above) to assess the involvement of different ion transport systems. Consecutive experiments (control followed by treatment experiments) were not performed due to the required length of such experiments and therefore, the current experimental design is associated with natural differences in cell-to-cell variability. Recovery rates were estimated from the compensatory slope after induced acidosis for the linear phase of recovery marked by the red lines in Fig. 2.

**Table 1:** Intracellular pH values from microfluorimetry experiments. Recovery pH_i_ under the presence of modified ASW/inhibitors at 60 min. Values presented as mean ± SEM

Baseline pH_i_	Alkalosis Compensation rate	Treatment after NH_3_/NH_4_^+^ pulse	Acidosis pH_i_	Recovery pH_i_	β	*N* (cells)	*N* (animals)
6.84 ± 0.04	− 0.0019 ± 0.0006	ASW	6.54 ± 0.04	6.96 ± 0.07	22.53 ± 1.26	11	6
6.72 ± 0.02	− 0.0033 ± 0.0005	Low Na^+^	6.36 ± 0.02	6.47 ± 0.03	19.6 ± 0.86	12	7
6.89 ± 0.06	− 0.0025 ± 0.0006	Low HCO_3_^−^	6.46 ± 0.03	6.73 ± 0.04	21.19 ± 1.35	10	5
6.81 ± 0.03	− 0.0022 ± 0.0003	20 μM EIPA	6.39 ± 0.05	6.61 ± 0.07	22.12 ± 2.3	17	5
6.89 ± 0.04	− 0.0021 ± 0.0002	1 mM ACZM	6.49 ± 0.04	6.83 ± 0.05	21.57 ± 1.04	29	7

### Buffer capacity

Buffer capacity (β) was estimated using the NH_3_/NH_4_ pulse as described by Boron (1977) and is expressed as Slykes (mM/pH unit). Concentrations of NH_4_^+^ in ASW were assumed to be negligible and nominally set to zero (Boron 1977). Following NH_3_/NH_4_^+^ pulse, β was calculated with the following formula:

$$\beta \, = \,\Delta \left[ {{\text{NH}}_{ 4}^{ + } } \right]/\Delta \left[ {{\text{pH}}_{\text{i}} } \right]$$

### Data analysis

All data were analysed using R (Version 3.3.2, R Development Core Team, R: http://www.R.org/. 2011). Data were tested for normality and homogeneity using Shapiro-Wilks test and Bartlett test, respectively. If assumption for normality was not met, data were transformed by applying Box-Cox transformations. To determine the ability of mantle epithelial cells to recover from an NH_3_/NH_4_^+^ induced acidosis, the alkalosis compensation rates, pH_i_ recovery rates and final pH_i_ (after 60 min) were tested for fixed effects of washout solution and the random effects of animals as replicate. A mixed effects model using the *lmer* function in the lmerTest package was applied and significant effects were determined using the ANOVA function. Post hoc analyses were performed via Tukey HSD tests. Data on Δ[H^+^] were analysed using a Kruskal–Wallis test followed by a Dunn’s posthoc test.

## Results

### Mantle epithelial cell culture

**Fig. 1:**
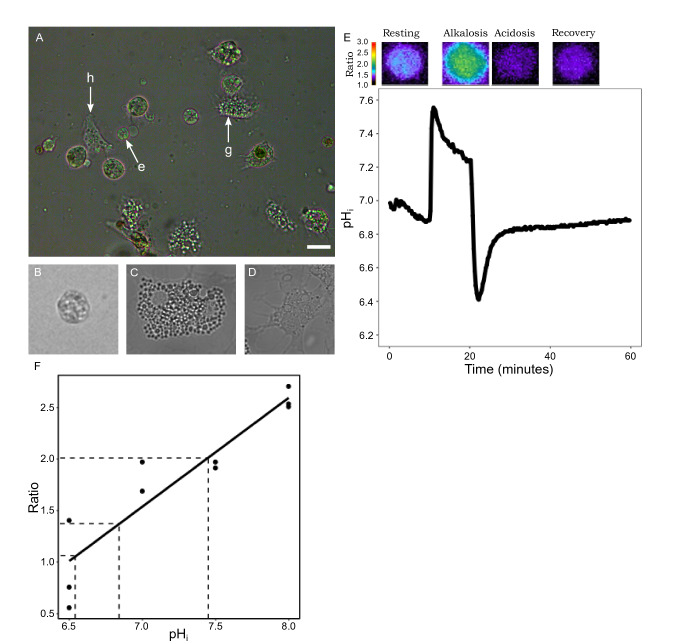
**A** Phase contrast image of mantle tissue cell culture in *Crassostrea gigas* depicting an epithelial cell (e), granular hemocytes (g) and hyalinocytes (h). Higher magnification images of all the three cell types labelled in (A): (**B**) epithelial cell (**C**) granular hemocytes (**D**) hyalinocyte. Scale bars: 20 μm in (A), 40 μm in (B), 30 μm in (C) and 50 μm in (D). **E** Fluorometric pH_i_ measurement in a mantle epithelial cell. **F** Calibration curve of BCECF-AM in mantle epithelial cells of *Crassostrea gigas* allowing the conversion of ratios to pH_i_ values. Dots represent individual cells

Following 24 h of cultivation, three typical cell populations were commonly observed around mantle explants, namely mantle epithelial cells, granular hemocytes and hyalinocytes (agranular hemocytes) (Fig. 1a–d). In addition, certain cultures contained the presence of spindle-like muscle cells (not shown). Cells were identified based on size, morphology and characteristic movement of the two hemocyte cell types as described previously (Awaji 1991; Gong et al. 2008). Although hemocytes have been linked to calcification in oysters (Mount et al. 2004; Ivanina et al. 2017), microfluorimetric measurements were not performed for these cell types due to a vesicular concentration of BCECF in addition to the cytosolic signal. Regions of the cell cultures containing higher abundance of the roundish, stationary epithelial cells were selected for microfluorimetric measurements.

### pH regulatory proteins in oyster mantle epithelia

**Fig. 2:**
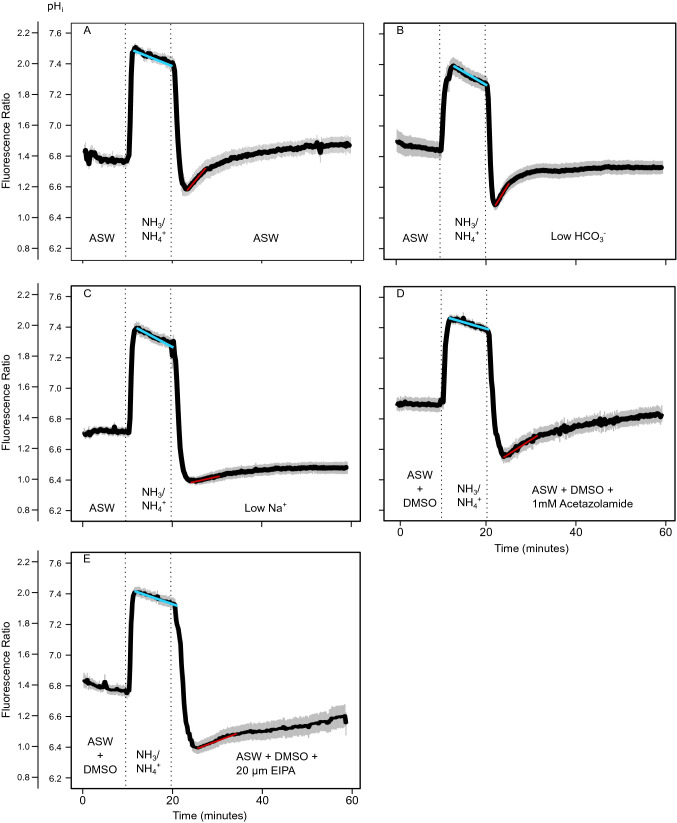
Acid-base regulatory capacities of mantle epithelial cells in *Crassostrea gigas* under the presence of various ASW solutions prepared according to Supplementary Table 1 and pharmacological inhibitors of specific cellular ion transport proteins. pHi recordings in the presence of **a** ASW (control recovery) **b** low HCO_3_^−^ **c** low Na^+^ **d** 1 mM acetazolamide and **e** 20 µM EIPA. Values are presented as mean ± SEM for various replicates as described in Table 1. Blue and red lines indicate slope of alkalosis and acidosis compensation respectively (color figure online)

**Fig. 3:**
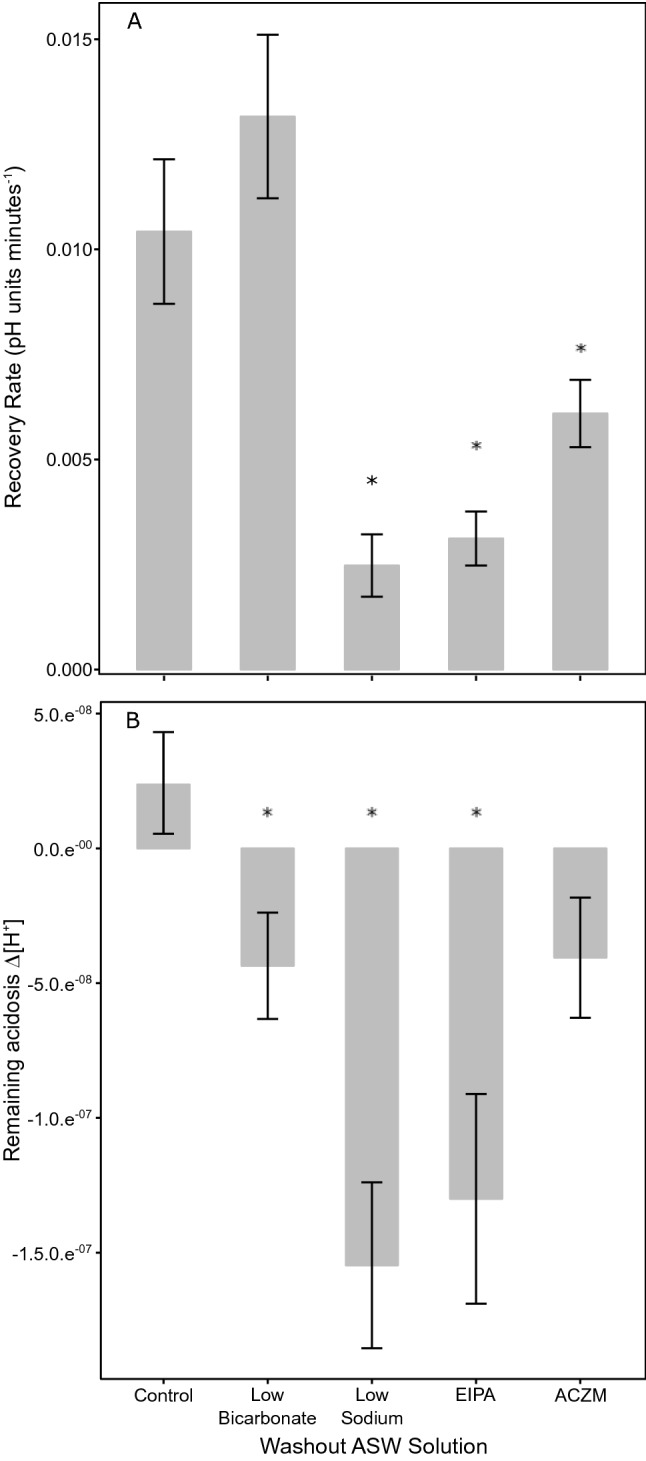
**a** Recovery rates of pH_i_ and **b** remaining difference in protons (Δ[H^+^]) during washout phase in *Crassostrea gigas* epithelial cells when exposed to various ASW solutions or pharmacological inhibitors of specific cellular ion transport proteins. Data are presented as mean ± SEM for various replicates as described in Table 1 and asterisks represent significant differences from control as tested by an ANOVA

Figure 1e shows a typical fluorometric measurement of one single mantle epithelial cell under resting conditions and after challenging the pHi by an ammonium pulse experiment. Cells where perfused with ASW (resting, control situation). Superfusion with 20 mM of the mild base NH_3_/NH_4_^+^ leads to alkalization (alkalosis) with slight counter-reaction of the cells (blue line), representing the alkalosis compensation rate, which was not significantly different between experiments (*p* > 0.05). This prepulse was followed by an acidosis phase upon wash-out of NH_3_/NH_4_^+^ leaving protons behind which have been produced as a result of alkalosis compensation. The following recovery phase (recovery) can be used to estimate proton extrusion and buffer capacity in cells (red lines in Fig. 2). Nigericin calibration was used to translate ratio into pH_i_ (Fig. 1f). *N* = 4–7 experiments were summarized for each of the measuring conditions in the following figures. An experiment was defined as cells isolated from *N* = 1 oyster. Figure 2a shows the control experiment where the wash-out was done by ASW (control). Cells maintained a resting pH_i_ of 6.84 ± 0.04. This pH_i_ was found to be stable for at least 180 min (Supplementary Fig. 2a, shown for one cell) and was used to establish cell culture status for further experiments. When cells were perfused with 20 mM NH_3_/NH_4_^+^, pH_i_ increased to a value of 7.45 ± 0.04. Removal of 20 mM NH_3_/NH_4_^+^ and perfusion with ASW induced an intracellular acidosis (pH_i_ 6.54 + 0.04) followed by gradual recovery in pH_i_ to 6.96 ± 0.07. A recovery rate (red line) of approx. 0.01 pH units/min was estimated (Fig. 2) for control conditions. Baseline pH_i_ for the different cell preparations varied under resting conditions and this variation may be biological or depend on seasonality. For this reason, we investigated rates of pH_i_ recovery following acidosis and Δ[H^+^] between resting and recovery phases. Figure 2b, c summarize the results for experiments where the washout of NH_3_/NH_4_^+^ was performed under low Na^+^ (B) and low bicarbonate (C) conditions where pH_i_ recovery in oyster mantle epithelial cells are significantly inhibited by modification of ASW in comparison to control experiments (ANOVA, F = 18.17, *p* < 0.05). Specifically, ASW treatments containing reduced Na^+^ or pharmacological inhibitors of the NHE (EIPA) resulted in significantly lower rates of pH_i_ recovery (Fig. 3, Tukey HSD, *p* < 0.05 respectively). To consider potential effects of DMSO, resting pH (prior to administration of inhibitors) between control and the two inhibitor experiments were examined and no significant differences were observed (*p* > 0.05, one-way ANOVA). Cell vitality following perfusion with these modified ASW solutions was confirmed by observing recovery to resting pH_i_ upon addition of control ASW containing comparable concentrations of DMSO (Supplementary Fig. 2b). In addition, rates of pH_i_ recovery were decreased when mantle epithelial cells were perfused with the carbonic anhydrase inhibitor, ACZM (Tukey HSD, *p* < 0.05). No significant effect on pH_i_ recovery rates was observed when mantle epithelial cells were perfused in ASW containing low HCO_3_^−^ (Tukey HSD, *p* > 0.05). However, final pH_i_ values following recovery period from the ammonium prepulse were significantly different in experiments where mantle epithelial cells were perfused with modified ASW solutions (ANOVA, F = 6.46, *p* < 0.05) where, experiments in the presence of low Na^+^ (Tukey HSD, *p* < 0.01) and EIPA (Tukey HSD, *p* < 0.01) revealed significantly lower pH_i_ at 60 min. Similarly, Δ[H^+^] were found to be significantly different (Kruskal–Wallis, *Χ*^2^ = 24.01, *p* < 0.05), where significant differences were found for the low HCO_3_^−^, low Na^+^ and EIPA washouts (Dunn’s Test, *p* < 0.05). Recovery rates ([H^+^]/minute) were − 3.67E − 08 ± 7.82E − 09, − 4.1E − 08 ± 4.33E − 09, − 3.98E − 09 ± 1.75E − 09, − 1.22E − 08 ± 2.53E − 09 and − 5.49E − 09 ± 9.65E − 10 for ASW, low bicarbonate, low Na^+^, EIPA and acetazolamide washouts respectively (Supplementary Fig. 4).

### Determination of buffer capacity (β)

We estimated β by perfusing mantle epithelial cells with 20 mM NH_3_/NH_4_^+^ followed by control ASW and observed a mean β value of 22.53 ± 1.26 mM/pH unit (n = 11 cells). Between experimental groups, within this short experimental period, calculated β was not different (Table 1).

## Discussion

Mollusc mantle epithelia have been long been studied to elucidate the biological control of calcification (Neff 1972; Joubert et al. 2010; Herlitze et al. 2018). It is crucial to understand the fundamental cellular acid–base regulatory pathways utilized by these cells to secrete calcified shells. The stable average pH_i_ of approx. 6.8 recorded by in vivo fluorescence imaging is comparable to those reported in other molluscan cells, including *C. gigas* hemocytes (Beckmann 2011; Michaelidis et al. 2005). Ammonium prepulse experiments demonstrate that oyster mantle epithelial cells have the capacity to recover from cellular acid load by a sodium dependent, EIPA-sensitive pathway. Additionally, we describe the buffer capacity (β) of calcifying mantle epithelial cells which may shed light on the ability of these cells to regulate pH_i_ when exposed to environmental hypercapnia and ongoing ocean acidification.

Using the ammonium prepulse technique, we observe a consistent cellular acidosis below control pH_i_ in *C. gigas* mantle epithelial cells, down to pH_i_ 6.54. This acidosis was followed by a recovery phase back to stable pH_i_ values around 6.96 (Table 1). Similar observations have been observed in other mollusc cell types including cardiac myocytes, oyster hemocytes and limpet oocytes (Ellington 1993; Vilain et al. 1993; Beckmann 2011). Our results demonstrate that the rates of pH_i_ recovery are significantly lower when cells are perfused with modified ASW solutions containing low Na^+^ or a pharmacological inhibitor, EIPA (Fig. 2, 3). The involvement of NHE proteins in pH_i_ regulation in mussel hemocytes and isolated mantle/gonad cells has been observed utilising pharmacological techniques (Kaloyianni et al. 2005; Banakou and Dailianis 2010). This group of ion-regulatory proteins has also previously been observed to exhibit upregulated mRNA expression in the mantle of the pearl oyster, *Pinctada fucata* during seawater acidification (Li et al. 2016). Within the genus *Crassostrea*, there are multiple genes encoding NHE transport proteins with similarity to cell membrane and intracellular (mitochondrial) isoforms in *Homo sapiens* and *Mus musculus*. However, an elementary molecular description for this group of antiporter proteins has been conducted in only one mollusc species, the giant clam, *Tridacna squamosa*, where NHE-3 isoforms have a predicted role in calcification (Hiong et al. 2017). This group of proteins has also been demonstrated to be crucial in the tolerance to seawater acidification in another mollusk, the squid, *Sepioteuthis lessoniana* (Hu et al. 2013, 2014). Taken together with the results from the present study, these observations indicate that NHE’s which are sensitive to EIPA are crucial to pH_i_ regulation in *C. gigas* mantle epithelial cells. Rates of pH_i_ recovery in *C. gigas* mantle epithelial cells are ca. 10 times slower than recovery rates observed in hemocytes within the same species (Beckmann 2011) but are comparable to recovery rates observed in cnidarian cells (Laurent et al. 2014), where NHE pathways have also been established to be important for pH_i_ regulation. Additionally, the compensation of induced alkalosis was very weak in oyster mantle epithelial cells in comparison to mammalian cells types (Bourgeois et al. 2018). This may be because these cells rarely experience such an extracellular alkalosis in the environment (seawater) or hemolymph and similar weak compensations to an ammonia induced alkalosis have also been observed in sea urchin larvae (Stumpp et al. 2012; Hu et al. 2018).

In addition to proton extrusion pathways mediated by NHEs and other proteins, cellular mechanisms of bicarbonate (HCO_3_^−^) transport can also play an important role in pH_i_ homeostasis. Our experiments indicate that modified ASW containing low HCO_3_^−^ has no significant effect on the rates of pH_i_ recovery and final pH_i_ values following induced cellular acidosis. However, it has to be mentioned here that little is known regarding the bicarbonate affinity of bicarbonate transporters in molluscs. Typically, bicarbonate affinities of acid/base proteins in marine invertebrates are matched to their environment and/or extracellular fluids (Tresguerres 2014). In the present study, the low HCO_3_^−^ solution was measured to contain 158 μmol kg^−1^ seawater HCO_3_^−^ and therefore, we cannot exclude the possibility of residual transport enabled by the minor fractions of HCO_3_^−^. In contrast to the results obtained on the role of extracellular HCO_3_^−^ in *C. gigas* pH_i_ regulation in mantle cells, significantly lower pH_i_ recovery rates in the presence of the carbonic anhydrase inhibitor, acetazolamide were observed. The enzyme carbonic anhydrase facilitates the reversible hydration of CO_2_ to HCO_3_^−^ and has long been suggested to be an important enzyme in mollusc shell forming tissue such as mantle epithelia (Medakovic 2000; Miyamoto et al. 2005; Yu et al. 2006; Aguilera et al. 2017). Seventeen genes encoding both extracellular and cytosolic isoforms of carbonic anhydrase proteins have been identified in the *C. gigas* genome (Zhang et al. 2012). Recent molecular cloning and characterisation of carbonic anhydrase II in *C. gigas* has revealed that this protein has highly conserved catalytic domains, is expressed in all tissues and its inhibition affects pH_i_ homeostasis (Wang et al. 2017). Further, in oysters, this carbonic anhydrase isoform is localised to the outer epithelia of mantle tissue and is observed to exhibit significant mRNA upregulation in response to CO_2_ exposure (Li et al. 2016; Wang et al. 2017).

Our results demonstrate that the activity of specific ion regulatory proteins such as NHEs and carbonic anhydrase are crucial for acid–base regulation. Interestingly, these proteins have also been associated with biomineralization (Medakovic et al. 2000; Zhang et al. 2012). Specifically, a suite of novel molecular studies lend support to the role of these carbonic anhydrases in acquisition of inorganic carbon during calcification (Wang et al. 2017; Koh et al. 2018; Chew et al. 2019). Additionally, it has been suggested that NHE proteins promote calcification by aiding in the removal of proton byproducts (Hiong et al. 2017; Cao-Pham et al. 2019). In bivalves, NHEs exhibit peaks in gene expression at the onset of larval calcification (Ramesh et al. 2019) and are also implicated in adult shell formation based on shell Na:Ca ratios (Zhao et al. 2017a, b). The concurrent activity of NHE and sodium/calcium exchange (NCX) proteins (Na^+^-dependent elevation of calcium) in mollusc calcification has not been studied. However, apical NCX proteins are suggested to be involved in calcium transfer across the oyster mantle epithelia (Sillanpaa et al. 2018) and whether Na^+^ exchange for calcium occurs following NHE mediated Na^+^ entry requires validation.

Although the role of ion transport is pivotal in pH_i_ homeostasis, cells may also minimize the effects of extracellular pH change through their cellular buffering capacity (β). The β of a specific cell type is related to the osmotic pressure, [HCO_3_^−^], glycolysis, sensitivity to pH and cation disturbances, the degree to which cells have the role of buffering the extracellular fluid and concentration of compounds containing histidine residues (Burton 1978; Abe 2000). In the present study, we used the NH_3_/NH_4_^+^ prepulse technique to determine β and observed a mean value of 22.53 Slykes, which is in the range reported for other molluscan cell types such as snail neurons and whelk radula muscle (25 Slykes, Thomas 1974 and 30 Slykes, Wiseman and Ellington 1989). However, oyster hemocytes within the same species have been observed to exhibit distinctly lower β (8 Slykes, Beckmann 2011). The relatively high β in *C. gigas* mantle epithelial cells may be consistent with the necessity of these cells to protect themselves from acid load during calcification, where protons are generated as byproducts.

In the context of global environmental change, there is little information on plasticity of pH_i_ regulation in these calcifying cells. One study that indirectly estimates pH_i_ in hemocytes suggests that *C. gigas* elevates pH_i_ upon CO_2_ exposure (Wang et al. 2016). Further, in response to seawater acidification, oysters have demonstrated an increased metabolic demand for NHEs which has been associated with increased proton extrusion (Stapp et al. 2018). Simultaneously, seawater acidification has been linked to an increased elimination of metabolic CO_2_ in oysters, a potential resilience mechanism (Stapp et al. 2018). However, without direct measurements, it is difficult to estimate the degree to which these organisms can respond to seawater pH reductions, particularly in the long term.

## Conclusion

We describe pH_i_ measurements in the mantle epithelial cells of *C. gigas* using an established microfluorimetric cell-imaging technique. The detected differences in pH_i_ regulatory capacities are a first step in identifying the functional cellular pathways for acid–base homeostasis of these cells. The decreased capacities of mantle epithelial cells to recover from an induced cellular acidosis as a result of exposure to low Na^+^ and all three pharmacological inhibitors indicate that Na^+^-driven ion transport pathways and carbonic anhydrases are an important component of the pH_i_ regulatory machinery in these cells. These findings are summarized in a first preliminary model of the pHi homeostasis machinery in mantle cells, highlighting the necessity for maintaining a Na^+^ gradient as driving force (NKA and NHE) and of CA in facilitating proton and bicarbonate generation (Supplementary Fig. 3). At present, several open questions remain regarding the physiology of molluscan calcification including the identification of bicarbonate transporters involved and the role of septate junctions in extracellular calcium transport.

## Electronic supplementary material

Below is the link to the electronic supplementary material.

Supplementary material 1 (PDF 3420 kb)

## Data Availability

Data can be accessed through PANGAEA database (10.1594/PANGAEA.920870).
