# Supplementary material for: Intracellular pH regulation in mantle epithelial cells of the Pacific oyster, Crassostrea gigas
Source: J Comp Physiol B. 2020 Aug 20;190(6):691–700. doi: 10.1007/s00360-020-01303-3 (PMC7520413; doi:10.1007/s00360-020-01303-3)
Supplement: Supplementary file 1 — Supplementary material 1 (PDF 3420 kb) [file 360_2020_1303_MOESM1_ESM.pdf]

Supplementary Figure 1. Photographs of the (**A**) fluorescence inverted microscope system and the (**B**) perfusion chambers (PC) used for experiments. Inflow (IF) and outflow (OF) tubes are tubes are displayed. Cover slips (CS) were attached to the chamber using a hydrophobic silicone grease and direction of flow is indicated by the arrows.

Supplementary Figure 2. Fluorometric pHi measurements in mantle epithelial cells of C. gigas demonstrating that (**A**) pHi could be maintained for a period of at least 180 minutes and that (**B**) pHi could be recovered following EIPA perfusion and was not affected by DMSO (i.e. cells still displayed vitality).

Supplementary Figure 3. A tentative ion transport model summarising the transport pathways following induced cellular alkalosis through the NH_3/NH_4^+ medium applied in experiments.

Supplementary Figure 4. Recovery rates given as proton concentration in mol per time during washout phase in *Crassostrea gigas* epithelial cells when exposed to various ASW solutions or pharmacological inhibitors of specific cellular ion transport proteins. Data are presented as mean ± SEM for various replicates as described in Table 1.

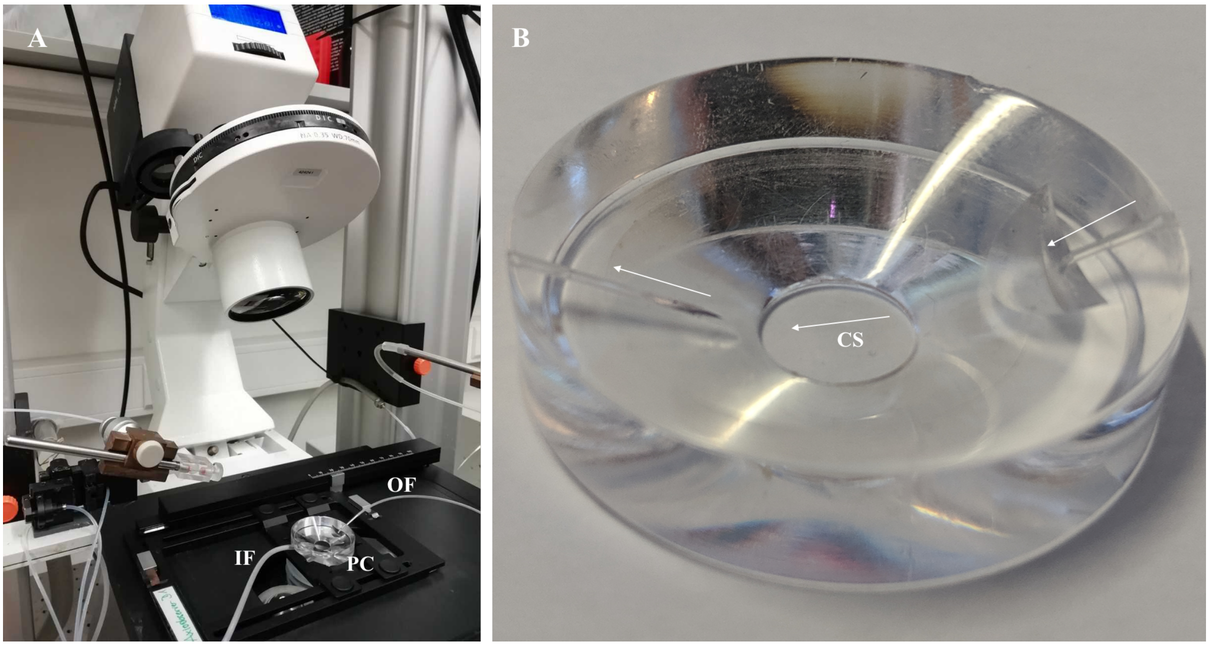
A
OF
IF
PC
B
CS

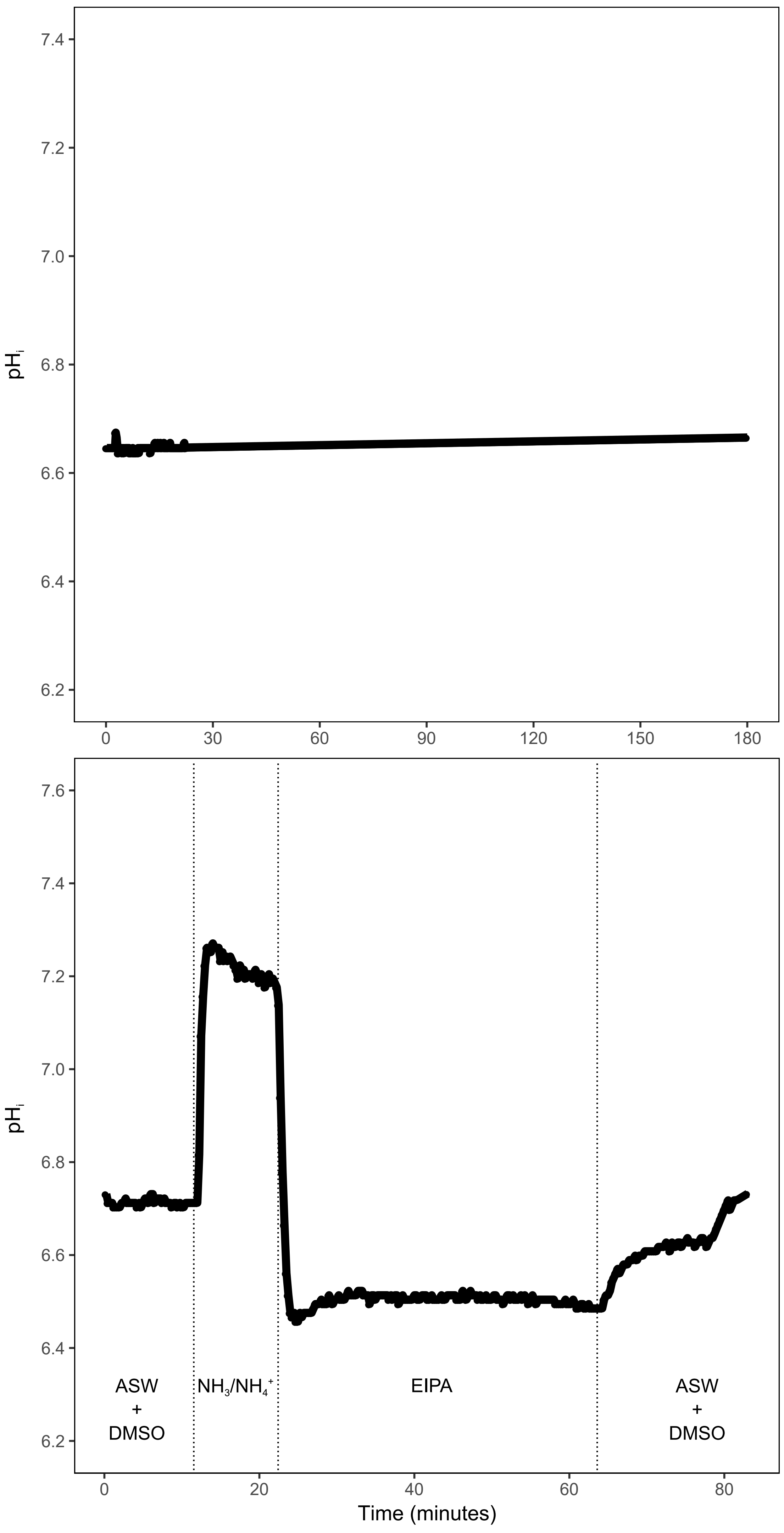

pHi
7.4
7.2
7.0
6.8
6.6
6.4
6.2
0
30
60
90
120
150
180
7.6
7.4
7.2
7.0
6.8
6.6
6.4
6.2
ASW
+
DMSO
NH3/NH4+
EIPA
ASW
+
DMSO
0
20
40
60
80
Time (minutes)

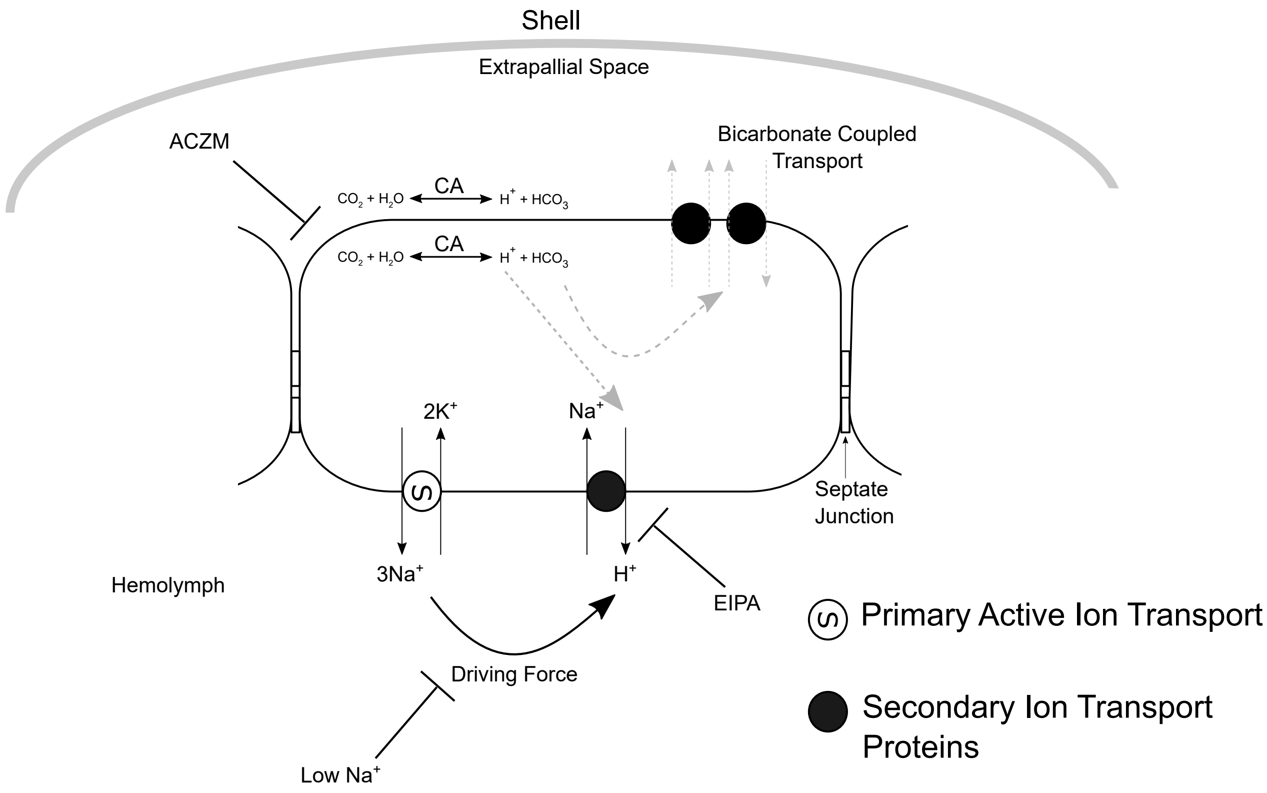

Shell
Extrapallial Space
ACZM
Bicarbonate Coupled Transport
CO2 + H2O
CA
H+ + HCO3
CO2 + H2O
CA
H+ + HCO3
2K+
Na+
Septate Junction
Hemolymph
3Na+
H+
EIPA
Driving Force
Low Na+
Primary Active Ion Transport
Secondary Ion Transport Proteins

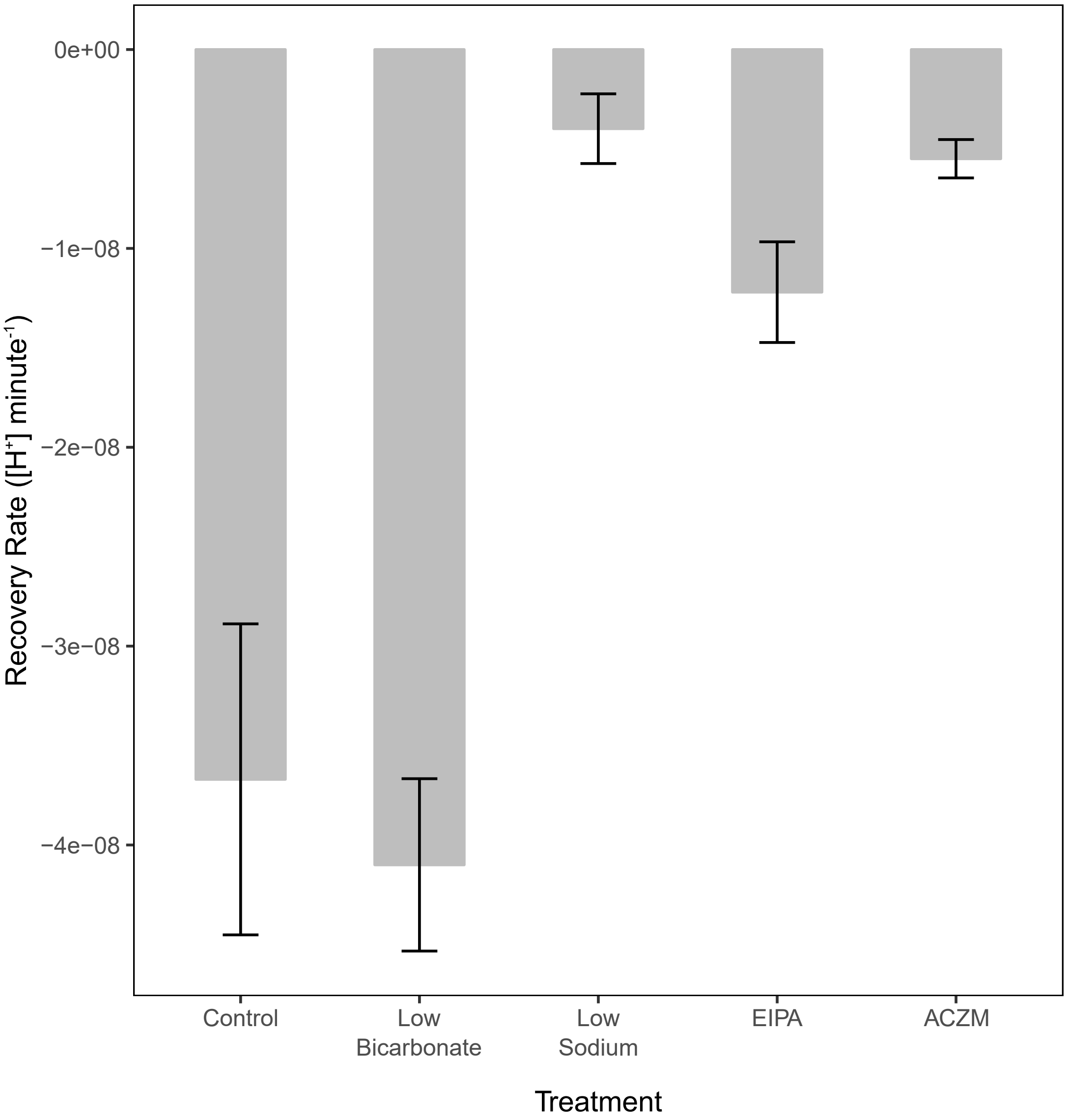

Recovery Rate ([H+] minute-1)
0e+00
−1e−08
−2e−08
−3e−08
−4e−08
Control
Low Bicarbonate
Low Sodium
EIPA
ACZM
Treatment

Supplementary Table 1. Composition of artificial seawater (ASW) solutions used according to Zeebe and Wolf-Gladrow (2001). Please note that pH was not measured for the calcium and magnesium free solution (prepared according to Gong et al 2008).

Reagent	ASW (mM)	Ca^{2+} & Mg^{2+} free (mM)	Calibration Buffer (mM)	20mM NH_3/NH_4^+ (mM)	Low Na^+ (mM)	Low HCO_3^- (mM)
NaCl	445	445	290	445	5	445
KCl	10	10	160	10	-	10
$CaCl_2.2H_2O$	10	-	10	10	10	10
$MgCl_2.6H_2O$	25.27	-	25.27	25.27	25.27	25.27
$MgSO_4.7H_2O$	28	-	28	28	28	28
$NaHCO_3$	2.35	2.35	2.35	2.35	2.35	-
NH_4Cl	-	-	-	20	-	-
Glucose	5	5	5	5	5	5
NMDG	-	-	-	-	443	-
HCl (1M)	-	-	-	-	443ml	-
HEPES	5	5	5	5	-	5
pH_{NBS}	8.001 ± 0.1	-	8.015 ± 0.16	8.108 ± 0.15	8.027 ± 0.1	8.1 ± 0.13
Osmolality (mOsm kg^{-1})	1104 ± 5	1107 ± 8	1100 ± 3	1110 ± 5	1098 ± 7	1106 ± 3

Supplementary Table 2. Composition of the cell culture medium used for *C. gigas* primary mantle cell culture. The recipe for the preparation of this medium was acquired from Gong et al (2008). Leibovitz-15 and Medium 199 are typically used for mammalian cell lines and therefore, to prepare a medium suitable for marine animal cells, their concentrations were elevated to 2x.

**Constituent**	**Final Concentration**
Leibovitz-15	2x
Medium 199	2x
NaCl	170.6 mM
Ascorbic Acid	40 μg/mL
Taurine	128.9 μg/mL
ATP	10 μg/mL
Lactalbumin Hydrolysate	400 μg/mL
HEPES	3.57 mg/mL
Penicillin	100 IU/mL
Streptomycin	100 μg/mL
Kanamycin	50 μg/mL
Nystatin	1 μg/mL
Fetal Bovine Serum	10%
